# Clinical, Molecular, and Immunological Characteristics of Scrub Typhus in Nepal

**DOI:** 10.1093/ofid/ofag390

**Published:** 2026-08-01

**Authors:** Anurag Adhikari, Carina Münch, Nora En-Nosse, Binod Rayamajhee, Gaurab Karki, Binod GC, Pardip Oli, Lea Fromm, Johanna Schwarz, Siddhesh Sapre, Jonas Mehl, Sagar Aryal, Dennis Tappe, Christian Keller

**Affiliations:** Department of Infection and Immunology, Kathmandu Research Institute for Biological Sciences, Lalitpur, Nepal; Infection and Immunity Program, La Trobe Institute of Molecular Science, Melbourne, Victoria, Australia; Institute of Virology, University Hospital Giessen and Marburg, Marburg, Germany; German Center for Infection Research (DZIF), site Giessen-Marburg-Langen, Germany; Institute of Virology, University Hospital Giessen and Marburg, Marburg, Germany; German Center for Infection Research (DZIF), site Giessen-Marburg-Langen, Germany; Department of Infection and Immunology, Kathmandu Research Institute for Biological Sciences, Lalitpur, Nepal; School of Optometry and Vision Science, Faculty of Medicine and Health, University of New South Wales, Sydney, New South Wales, Australia; Faculty of Medicine, Macquarie University, Sydney, New South Wales, Australia; Department of Infection and Immunology, Kathmandu Research Institute for Biological Sciences, Lalitpur, Nepal; Department of Infection and Immunology, Kathmandu Research Institute for Biological Sciences, Lalitpur, Nepal; Center of Molecular and Cellular Oncology, Yale University, New Haven, Connecticut, USA; Mount Sagarmatha Polyclinic and Diagnostic Centre, Nepalgunj, Nepal; Institute of Clinical Microbiology and Hygiene, University Medical Center Regensburg, Regensburg, Germany; Department of Data Science in Biomedicine, Philipps University Marburg, Marburg, Germany; Institute of Virology, University Hospital Giessen and Marburg, Marburg, Germany; Institute of Virology, University Hospital Giessen and Marburg, Marburg, Germany; Department of Infection and Immunology, Kathmandu Research Institute for Biological Sciences, Lalitpur, Nepal; Bernhard Nocht Institute for Tropical Medicine, Hamburg, Germany; Institute of Virology, University Hospital Giessen and Marburg, Marburg, Germany; German Center for Infection Research (DZIF), site Giessen-Marburg-Langen, Germany; Institute of Clinical Microbiology and Hygiene, University Medical Center Regensburg, Regensburg, Germany

**Keywords:** neglected tropical diseases, Nepal, *Orientia tsutsugamushi*, qPCR, scrub typhus

## Abstract

In a serologically diagnosed Nepalese scrub typhus cohort (n = 77), gastrointestinal symptoms were common. We assessed *Orientia tsutsugamushi* DNA in serum and performed TSA56 genotyping. *Orientia* DNA was detected in 10 of 77 samples (12%). TSA56 sequencing identified 4 genotypes. Polymerase chain reaction positivity was associated with diarrhea, groin lymphadenopathy, and stronger Th1-type cytokine responses.

## Background

Scrub typhus is a neglected tropical infectious disease caused by the obligate intracellular bacterium *Orientia tsutsugamushi (Ot)* and has emerged as an important mite-borne zoonosis in the Asia-Pacific region. High *Ot* DNA loads in blood samples have been associated with severe disease [1, 2] and with type 1 immune responses during the acute phase [3–6]. Molecular data from Nepal, however, remain limited, especially with correlation to clinical presentation. Moreover, the diversity of human-pathogenic *Ot* from Nepal is poorly defined. While one study suggested that Nepalese strains cluster together and away from other Asian strains [7], Münch et al demonstrated that Nepalese TSA56 sequences have high homologies to sequences obtained from Vietnam, Thailand, Taiwan, and India [5]. Conversely, individual TSA56 sequences from Bhutan were similar to recently published Nepalese strains [8].

The present study was performed to obtain clinical and molecular data (ie, quantitative DNA loads and strain-specific sequences) from Nepalese scrub typhus patients and to correlate these to reported clinical symptoms. Also, systemic cytokine concentrations were measured and correlated to polymerase chain reaction (PCR) positivity.

## MATERIALS AND METHODS

### Patient Recruitment and Scrub Typhus Diagnosis

Individuals with current or recent (>38.0°C within 72 hours, self-reported) fever (n = 82; median age, 53 years [IQR, 37–61 years]; 37% female), diagnosed with scrub typhus using the Scrub Typhus Detect immunoglobulin M (IgM) rapid test (InBios, Seattle, WA, USA) and negative for dengue and malaria, were included from Mount Sagarmatha Polyclinic and Diagnostic Centre/Bheri Zonal Hospital and Dhading District Hospital (April 2016–January 2017). Pregnant women and children <18 years of age were excluded. Clinical symptoms were recorded using a standardized questionnaire. All patients received outpatient care and were not hospitalized. Sufficient material for PCR and cytokine analyses was available for 77 samples. For immunological assays, previously reported values from healthy Nepalese blood donors (n = 50; median age, 53 years [interquartile range {IQR}, 44–61 years]; 45% female) served as a reference [5, 9]. All participants provided written informed consent.

### Quantification of Cytokines by Multiplex Bead Array

For measurement of interleukin 6 (IL-6), interleukin 10 (IL-10), interferon gamma (IFN-γ), and tumor necrosis factor alpha (TNF-α), serum samples were tested using the Human Th cytokine panel (BioLegend, San Diego, CA, USA) according to the manufacturer’s instructions, with minor protocol alterations [5].

### Real-time Quantitative PCR and Conventional PCR

Total nucleic acids were extracted from diluted patient sera (EZ1 Virus Mini Kit v2.0; Qiagen, Hilden, Germany). Sera (50 µL) were diluted with 350 μL phosphate-buffered saline for a total extraction volume of 400 µL (ratio 1:8). Elution volume was 60 µL.

For molecular detection of *Ot*, we used a 16S rRNA gene-specific quantitative PCR (qPCR) [1], with a modified annealing temperature of 56°C. Oligonucleotide sequences were: OT3F (5′-CCCATCAGTACGGAATAACA-3′), OT1R (5′-CTCTCAGACCAGCTACAGATCACA-3′), and OT_16S_probe (5′-6FAM-TAAGTGCTAATACCGTATGCCCTCTA-BHQ1-3′; Biomers, Tübingen, Germany). The human RNAseP gene was utilized as internal control (RNAseP_forward: 5′-AGATTTGGACCTGCGAGCG-3′, RNAseP_reverse: 5′-GAGCGGCTGTCTCCACAAGT-3′, RNAseP_probe660: 5′-Cy5-TTCTGACCTGAAGGCTCTGCGCGT-Q3-3′), as described previously [10]. Quantification of 16S genomic equivalents was performed using a standard curve constructed from a pCRTM2.1 plasmid vector containing the target sequence (Thermo Fisher Scientific, Waltham, MA, USA). For confirmation of 16S-positive samples, a TSA47 gene-specific qPCR was used [11]. Primer sequences were OtsuFP630_forward: 3′-AACTGATTTTATTCAAACTAATGCTGCT, OtsuRP747_reverse: 3′-TATGCCTGAGTAAGATACRTGAATRGAATT-5′, OtsuPR665_probe: 3′-6FAM-TGGGTAGCTTTGGTGGACCGATGTTTAATCT-BMN530-5′. Reactions were run on a LightCycler 480 in a volume of 20 µL using the LightCycler Multiplex 5× DNA Master (both Roche, Mannheim, Germany). Partial sequences of the TSA56 gene were amplified by conventional PCR according to Mahajan et al [12], using a Thermocycler GenAmp2700 (Applied Biosystems). Primer sequences were Otsu_F_56kD_forward: 5′-AATTGCTAGTGCAATGTCTG-3′, Otsu_R_56kD_reverse: 5′-GGCATTATAGTAGGCTGAG-3′. Six amplicons were sequenced bidirectionally (Seqlab Microsynth, Göttingen, Germany) and deposited in the National Center for Biotechnology Information (NCBI) GenBank (accession numbers ON361136-ON361141).

### Statistical Analysis and Computational Methods

Statistical analyses were performed using GraphPad Prism (version 10.4.1). Phylogenetic analysis involved a ClustalW multiple alignment with sequences retrieved from the NCBI database in Geneious software (version 11.1.5), followed by constructing a neighbor-joining tree (Tamura–Nei model).

### Ethics Statement

The study protocol was approved by the Nepal Health Research Council (no. 292/2015) and the Institutional Review Board of Philipps University Marburg (no. 23/21).

## RESULTS

The most frequent clinical finding in IgM-positive patients was fever, which was found in 62 of 77 (80.5%) participants, followed by chills (74.0%), nausea (72.7%), and headache (66.2%) (Table 1). Participants also reported vomiting (57.1%), abdominal discomfort (57.1%), and diarrhea (31.2%), indicating gastrointestinal (GI) involvement in a substantial proportion of individuals. An eschar was identified in 13 of 77 (16.9%) patients, and 24 of 77 (31.2%) patients presented with generalized lymphadenopathy.

**Table 1.** Clinical Data of 77 Nepalese Patients With Scrub Typhus and Correlation With Quantitative Polymerase Chain Reaction Results

Clinical Data	Total (N = 77)	qPCR Positive (n = 10)	qPCR Negative (n = 67)	Odds Ratio (95% CI)	*P* Value (Fisher's Exact)
Febrile sensation before presentation	35 (45.5%)	8 (80%)	27 (40.3%)	5.9 (1.2–28.8)	.**037**
Chills	57 (74%)	9 (90%)	48 (71.6%)	3.6 (.5–40.9)	.439
Headache	51 (66.2%)	8 (80%)	43 (64.2%)	2.2 (.5–11.0)	.480
Nausea	56 (72.7%)	5 (50%)	51 (76.1%)	0.3 (.1–1.2)	.125
Vomiting	44 (57.1%)	5 (50%)	39 (58.2%)	0.7 (.2–2.5)	.737
Abdominal discomfort	44 (57.1%)	8 (80%)	36 (53.7%)	3.4 (.7–16.9)	.174
Diarrhea	24 (31.2%)	7 (70%)	17 (25.4%)	6.9 (1.5–25.8)	.**008**
Fever at presentation (>38.0°C)	62 (80.5%)	9 (90%)	53 (79.1%)	2.4 (.4–27.8)	.676
Tachycardia (heart rate >100 beats/min)	3 (3.9%)	0%	3 (4.5%)	0.0 (.0–8.0)	>.999
Jaundice	9 (11.7%)	0%	9 (13.4%)	0.0 (.0–3.0)	.596
Crackles	9 (11.7%)	1 (10%)	8 (11.9%)	0.8 (.1–5.8)	>.999
Muscle tenderness	7 (9.1%)	2 (20%)	5 (7.5%)	3.1 (.5–16.8)	.223
Petechiae in mouth	2 (2.6%)	0%	2 (3%)	0.0 (.0–14.9)	>.999
Petechiae in axilla	4 (5.2%)	1 (10%)	3 (4.5%)	2.4 (.1–17.2)	.434
Petechiae on abdomen	1 (1.3%)	0%	1 (1.5%)	0.0 (.0–60.3)	>.999
Petechiae on back	1 (1.3%)	0%	1 (1.5%)	0.0 (.0–60.3)	>.999
Petechiae (other)	3 (3.9%)	1 (10%)	2 (3%)	3.61 (.2–32.7)	.345
Hepatomegaly	8 (10.4%)	1 (10%)	7 (10.4%)	1.0 (.1–7.2)	>.999
Lymphadenopathy (generalized)	24 (31.2%)	5 (50%)	19 (28.4%)	2.5 (.7–9.1)	.270
Lymphadenopathy (neck)	12 (15.6%)	2 (20%)	10 (14.9%)	1.4 (.3–6.3)	.650
Lymphadenopathy (axilla)	5 (6.5%)	0%	5 (7.5%)	0.0 (.0–5.1)	>.999
Lymphadenopathy (groin)	6 (7.8%)	3 (30%)	3 (4.5%)	32.0 (5.8–137.4)	**<**.**0001**
Skin wound visible (open)	1 (1.3%)	0%	1 (1.5%)	0.0 (.0–60.3)	>.999
Eschar	13 (16.9%)	2 (20%)	11 (16.4%)	1.3 (.3–5.8)	.674

Shown are the numbers of individuals presenting with the indicated symptoms as assessed by the questionnaire (clinical items; n = 77), with respect to qPCR results. N indicates total number of patients. Data are presented as No. (%) unless otherwise indicated. *P* values were calculated using the two-sided Fisher’s exact test.

Abbreviations: CI, confidence interval; qPCR, quantitative polymerase chain reaction.

To test cell-free serum samples for circulating *Ot* DNA, we conducted a qPCR targeting an *Ot*-specific 16S rRNA gene sequence [1]. *Ot* DNA was detectable in 10 of 77 analyzed samples (13.0%); 67 of 77 samples (87.0%) were negative by qPCR. The concentration of genome equivalents ranged between 1.4 × 10^2^ and 2.6 × 10^4^ copies/mL (median, 7.7 × 10^2^ [IQR, 2.4 × 10^2^–1.7× 10^4^]). Nine of 10 (90.0%) *Ot* DNA-positive samples were confirmed by TSA47 gene-specific qPCR.

To explore the clinical relevance of qPCR positivity in scrub typhus patients, we analyzed the frequency of *Ot* DNA detection in serum in individuals with and without reported symptoms. Notably, groin lymphadenopathy was most strongly associated with qPCR positivity (Table 1), with an odds ratio (OR) of 32.0 (95% confidence interval [CI], 5.8–137.4; *P* < .0001), while lymphadenopathy in other localizations did not differ significantly between both groups. Diarrhea (OR, 6.9 [95% CI, 1.5–25.8]; *P* = .008) and febrile sensation before presentation (OR, 5.9 [95% CI, 1.2–28.8]; *P* = .037) were also significantly associated with qPCR positivity, although these associations were weaker.

**Figure 1.**
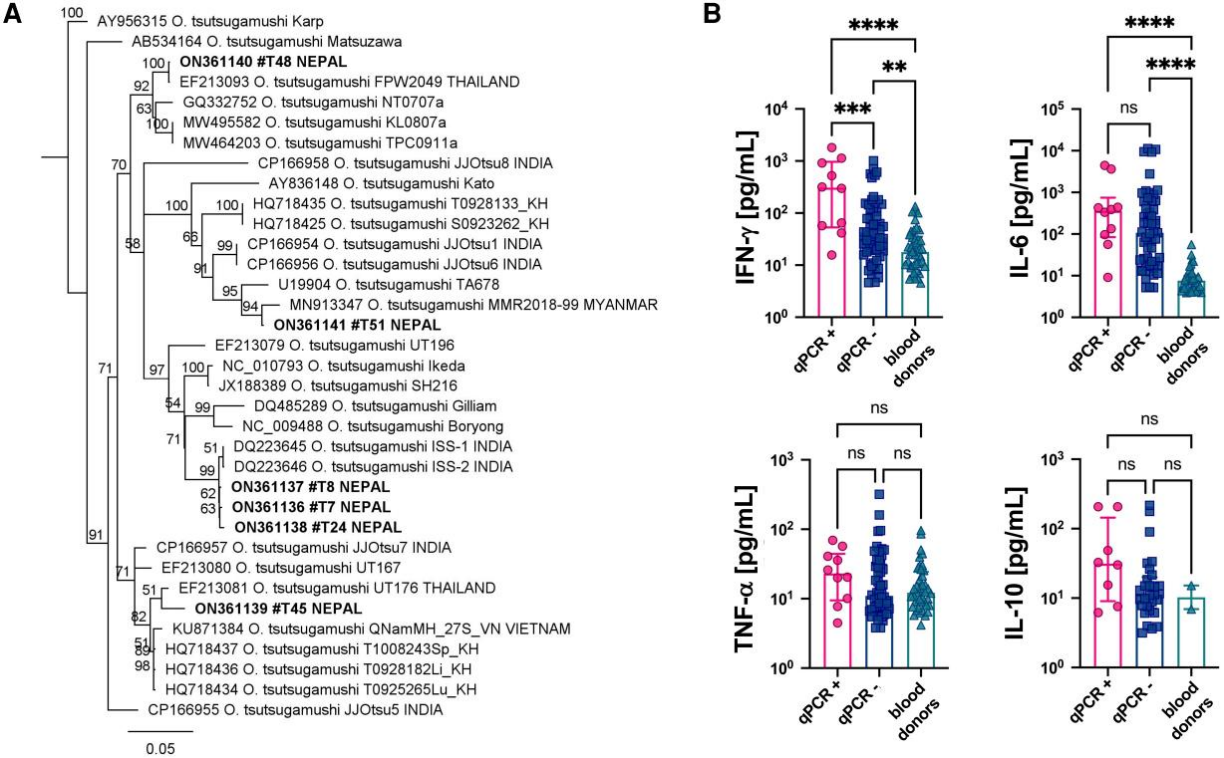
Phylogenetic characterization of *Orientia tsutsugamushi* (*Ot*) and serum cytokine concentrations in Nepalese individuals with scrub typhus. *A*, TSA56 gene sequences from 6 patient samples were trimmed from primer binding sites and aligned with the 3 or 4 closest matches from the National Center for Biotechnology Information BLAST database; the *Ot* prototype strains Karp, Kato, Gilliam, Ikeda, and Boryong; and 5 recently sequenced strains from India [13], using the Geneious alignment algorithm. A phylogenetic tree was constructed using the Tamura–Nei model and the neighbor-joining method in Geneious software. *B*, Concentrations of interferon gamma (IFN-γ), interleukin 6 (IL-6), tumor necrosis factor alpha (TNF-α), and interleukin 10 (IL-10) in serum samples were quantified by LegendPlex multiplex bead-based immunoassay. Shown are the measurable concentrations from patients positive or negative by 16S quantitative polymerase chain reaction (qPCR) and Nepalese blood donor controls. ns, not significant; **P < .01, ***P < .001, ****P < .0001 by pairwise comparison with Wilcoxon rank-sum test and Holm’s P value adjustment method.

In order to determine the *Ot* genotype, we amplified a proportion of the TSA56 gene from qPCR-positive samples. In 9 of 10 samples, a TSA56 product was obtained. Sequencing succeeded for 6 of 9 amplicons (362–411 bp). Phylogenetic analysis of these 6 sequences placed Nepalese *Ot* strains into 4 clusters (Figure 1*A*): 3 sequences were highly similar and grouped with reference strains from India, whereas the remaining sequences grouped with strains from Thailand, Myanmar, Vietnam, or India.

We next explored the cytokine response associated with qPCR positivity in scrub typhus patients, using a multiplex array. Levels of IFN-γ, but not IL-6, TNF, and IL-10, were significantly elevated in qPCR-positive compared to qPCR-negative patients (Figure 1*B*), suggesting a stronger Th1-type response, while the overall proinflammatory cytokine profile remained comparable. These findings support the idea that qPCR positivity reflects active infection characterized by a pronounced Th1 response.

## DISCUSSION

This study sheds new light on the clinical profile of scrub typhus and the human-pathogenic *Ot* strains circulating in Nepal. To our knowledge, it is the first report from Nepal that demonstrates an association between clinical symptoms (ie, lymphadenopathy and diarrhea) and the *Ot* DNA load in the first diagnostic blood sample.

Fever was documented at presentation in 80.5% of patients, lower than reported in some previous studies (often >90% [14]). In the remaining patients, a history of fever prior to presentation was based on self-report, which is a potential limitation of this study. The 16S qPCR utilized here detected *Ot* DNA in 13% of acute cases—which is below the 39.1%–52% reported elsewhere [1, 5]. This lower ratio likely reflects lower analytical sensitivity of serum and the small sample volume used; whole-blood or buffy coat samples are generally more sensitive [15, 16]. While the sample volume represents another potential limitation, the observed associations with clinical manifestations were driven by high bacterial DNA loads, which are reliably detected at the volume used.

Our study found a high proportion of individuals with upper GI symptoms during scrub typhus from Nepal, including nausea, vomiting, and abdominal discomfort, which is consistent with reports from Thailand, Taiwan, and China [17–19]. Upper GI tract endoscopy in acutely ill scrub typhus patients has shown superficial mucosal hemorrhage, erosions, ulcers, or vascular bleeding. Histological analyses of these lesions have demonstrated features of small vessel vasculitis that correlate with clinical severity, suggesting a possible contribution to the pathogenesis of upper GI symptoms [20].

Diarrhea, typically regarded as a lower GI manifestation, was another frequent symptom. The association of qPCR positivity with diarrhea in scrub typhus is a novel finding. It may reflect a higher systemic DNA burden in individuals with intestinal involvement. Previous studies have shown higher *Ot* DNA loads in people with higher levels of clinical severity [2], particularly with pneumonia [21], or in patients with longer duration of symptoms, and in those who died from scrub typhus [1]. Other factors influencing the *Ot* DNA load during acute infection are, for example, the host’s genetic background, the *Ot* strain involved, or the presence of an eschar [1, 22].

The higher odds of groin lymphadenopathy among qPCR-positive patients in our study may equally reflect a greater systemic bacterial burden in these individuals, as blood qPCR positivity is itself linked to more severe infection [1]. We also showed that qPCR-positive patients had higher serum IFN-γ levels compared to the qPCR-negative group, suggesting a stronger induction of type 1 immunity in cases with bacteremia, which is consistent with the reported correlation between serum IFN-γ and *Ot* DNA [5].

Regarding strain diversity, TSA56 sequences clustered into 4 genotypes. Although based on limited numbers, this pattern does not support a distinct Nepalese clade [7], but instead suggests considerable genetic heterogeneity among Nepalese *Ot* strains and close phylogenetic relationships to those in neighboring countries.

In conclusion, our study highlights GI symptoms as a frequent manifestation of scrub typhus in Nepal, with diarrhea being associated with positive serum qPCR. This finding may have translational relevance, as diarrhea could serve as a simple clinical marker of higher systemic *Ot* burden and increased likelihood of molecular detectability. The concomitant association of serum qPCR positivity with elevated IFN-γ links bacterial DNAemia and Th1-type immune activation, suggesting a shared axis of disease activity and severity. Finally, the diversity of Nepalese human-pathogenic *Ot* strains highlights the need to consider local strain variability in epidemiological surveillance and future vaccine development.
